# Beyond cell-cell contact: therapeutic potential of Eph signaling in central nervous system tumors

**DOI:** 10.3389/fnmol.2025.1658651

**Published:** 2025-10-22

**Authors:** Fernanda Cristina Poscai Ribeiro, Moisés Willian Aparecido Gonçalves, Aleff Mascarenhas Silva, Tayná Figueiredo Maciel, Reydson Alcides de Lima-Souza, João Figueira Scarini, Gary Chris Fillmore, Erika Said Abu Egal

**Affiliations:** ^1^Department of Internal Medicine, Western São Paulo University (UNOESTE), Medical School, Guarujá, São Paulo, Brazil; ^2^Department of Oral Diagnosis, Piracicaba Dental School, Universidade Estadual de Campinas (UNICAMP), Piracicaba, São Paulo, Brazil; ^3^Department of Pathology, School of Medical Sciences, Universidade Estadual de Campinas (UNICAMP), Campinas, São Paulo, Brazil; ^4^Biorepository and Molecular Pathology, Huntsman Cancer Institute (HCI), University of Utah (UU), Salt Lake City, UT, United States

**Keywords:** Eph receptors, ephrins, glioma, glioblastoma, medulloblastoma, meningioma

## Abstract

Eph receptor tyrosine kinases and their membrane-bound ephrin ligands constitute a unique bidirectional signaling system that orchestrates cell adhesion, migration, proliferation, and vascular patterning, processes frequently co-opted in malignancy. We conducted an integrative review of preclinical models and clinical cohorts to delineate Eph/ephrin expression landscapes and evaluate functional outcomes in central nervous system neoplasms. In gliomas, particularly glioblastoma multiforme, overexpression of EphA2 and EphA3 correlates with higher tumor grade and increased invasiveness. Conversely, ephrin-A1 and ephrin-A5 exhibit tumor-suppressive properties by promoting receptor internalization and degradation, thereby inhibiting glioma cell proliferation and migration. In medulloblastoma, elevated expression of EphB1 and EphA4 is associated with enhanced angiogenesis and migratory capacity, contributing to tumor progression. In meningiomas, aberrant activation of EphA2 and EphB1 promotes proliferation through engagement with mTOR and ERBB3 signaling pathways. Emerging therapeutic strategies, including ligand-targeted cytotoxins, selective kinase inhibitors, chimeric antigen receptor T cells, and ephrin-based immunomodulators, demonstrate potent anti-tumor efficacy in preclinical settings, highlighting the translational potential of targeting the Eph/ephrin axis. The dualistic nature of Eph/ephrin signaling underscores its translational promise as both a biomarker framework and a precision-guided therapeutic target. Combinatorial receptor-ligand modulation strategies may advance the treatment of central nervous system malignancies by exploiting the context-dependent roles of Eph/ephrin interactions.

## 1 Introduction

Eph receptors and their ligands, ephrins, constitute the largest family of receptor tyrosine kinases (RTKs; Hirai et al., 1987). The name “Eph” derives from the erythropoietin-producing hepatocellular carcinoma cell line from which the first member of this receptor family was isolated. Although this origin is etymologically descriptive, “Eph” is not a functional acronym and is now used generically to refer to this receptor family.

Eph receptors are key regulators of several cellular processes, including cell adhesion, migration, and proliferation. A unique feature of the Eph/ephrin system is its bidirectional signaling: forward signaling occurs through phosphorylation-dependent mechanisms in Eph-expressing cells, while reverse signaling is activated in ephrin-expressing cells (Kullander and Klein, 2002; Surawska et al., 2004).

These receptors are subdivided into two main subclasses, EphA and EphB, which generally bind to ephrin-A and ephrin-B ligands, respectively (Papadakos et al., 2022). Under physiological conditions, Eph/ephrin interactions are essential for developmental processes, including axon guidance, synapse formation, and vascular remodeling (Lisabeth et al., 2013).

In pathological contexts such as cancer, Eph/ephrin signaling assumes a dual role. Some members of this family exhibit tumor-suppressive properties, while others are associated with enhanced tumor growth, invasion, metastasis, and unfavorable prognosis (Anderton et al., 2021). This dichotomy reflects the context-dependent nature of Eph/ephrin functions across different tumor types.

In tumors of the central nervous system (CNS), the Eph/ephrin system has emerged as a critical player in tumorigenesis (Ostrom et al., 2022; Liu et al., 2007; Lertsumitkul et al., 2024). This article provides a comprehensive overview of the role of Eph receptors and ephrins in cancer, with a particular emphasis on CNS tumors. It highlights the molecular mechanisms involved, their clinical significance, and the growing potential for therapeutic interventions targeting this signaling axis.

## 2 Methodology

The objective of the search was to identify in both clinical and preclinical investigations that assessed the role of Eph receptors and ephrins in the initiation, progression, or therapeutic targeting of CNS neoplasms. The electronic database of PubMed was systematically searched from its inception to May 2025 using a predefined search strategy without language limitation. The following search was applied:

(ephrin^*^ *OR “Eph receptor” OR “Eph receptors” OR “Ephrin receptor” OR “Ephrin receptors”) AND (“central nervous system tumor” OR “CNS tumor” OR “CNS neoplasm” OR “brain tumor” OR “spinal cord tumor” OR glioma OR glioblastoma OR astrocytoma OR oligodendroglioma OR ependymoma OR medulloblastoma OR meningioma OR schwannoma OR “ependymal tumor” OR “choroid plexus tumor” OR “primary CNS lymphoma”)*

In addition, papers that exclusively provided narrative overviews were excluded. The initial screening of titles and abstracts was performed to exclude irrelevant publications. Full-text articles were subsequently reviewed to confirm eligibility. To enhance methodological rigor and reduce selection bias, the process of study identification and inclusion was independently supervised by two authors.

## 3 Overview

### 3.1 Tumors of the central nervous system

Tumors of the CNS encompass a diverse group of neoplasms that differ markedly in histopathological features, molecular profiles, clinical behavior, and prognosis (WHO Classification of Tumours Editorial Board, 2021). Although CNS tumors represent only a small percentage of all malignancies, they account for a disproportionately high burden of neurological (Bray et al., 2024; Filho et al., 2025). According to the GLOBOCAN 2022 estimates, there were approximately 321,731 new cases of brain and CNS tumors worldwide, accounting for about 1.6% of all new cancer diagnoses. These malignancies led to 248,500 deaths, representing roughly 2.6% of global cancer-related mortality. Importantly, the global burden of CNS tumors is expected to increase substantially over the coming decades, with projections estimating 457,000 new cases and 356,000 deaths by 2040. This growing incidence is largely attributed to population growth and aging, particularly in low- and middle-income countries (International Agency for Research on Cancer, 2022).

The fifth edition of the World Health Organization (WHO) classification of CNS tumors introduced a more refined, integrated diagnostic approach that combines histological features with molecular profiling (WHO Classification of Tumours Editorial Board, 2021). This paradigm shift has greatly enhanced the precision of tumor categorization, allowing for more accurate prognostication and treatment stratification. Tumors are now divided into distinct groups, including gliomas, glioneuronal and neuronal tumors, choroid plexus tumors, embryonal tumors, pineal tumors, cranial and paraspinal nerve tumors, meningiomas, mesenchymal non-meningothelial tumors, melanocytic tumors, hematolymphoid tumors, germ cell tumors, sellar region tumors, and metastases to the CNS (WHO Classification of Tumours Editorial Board, 2021; Table 1).

**Table 1:** Classification of CNS tumors.

**Tumor group**	**Description**	**Typical location**	**Prognosis**	**Subtypes**
Gliomas, glioneuronal tumors, and neuronal tumors	Most common and varied tumors of CNS parenchyma, derived from glial and neuronal cells	Brain parenchyma	Variable: from indolent to highly aggressive	- Adult-type diffuse gliomas (astrocytoma IDH-mutant, oligodendroglioma IDH-mutant and 1p/19q-codeleted, glioblastoma IDH-wildtype) - Pediatric-type diffuse low-grade gliomas - Pediatric-type diffuse high-grade gliomas - Circumscribed astrocytic gliomas - Glioneuronal and neuronal tumors - Ependymal tumors (ependymomas, classified by site and molecular features)
Choroid plexus tumors	Tumors arising from choroid plexus epithelial cells, with marked epithelial characteristics	Ventricles (mainly lateral and fourth ventricles)	Variable; generally better prognosis in children	- Choroid plexus papilloma - Atypical choroid plexus papilloma - Choroid plexus carcinoma
Embryonal tumors	Highly aggressive, primitive neuroectodermal tumors, mostly in children	Cerebellum, brainstem, and other CNS sites	Generally poor prognosis	- Medulloblastoma - Atypical teratoid/rhabdoid tumor - Embryonal tumor with multilayered rosettes
Pineal tumors	Tumors originating from pineal gland cells, often with neuroendocrine features	Pineal region	Variable, depending on tumor type	- Pineocytoma - Pineoblastoma - Germ cell tumors affecting the pineal region
Cranial and paraspinal nerve tumors	Tumors of nerve sheaths of cranial and spinal nerves, mostly benign	Along cranial nerves and spinal nerve roots	Generally favorable	- Schwannoma - Neurofibroma - Malignant peripheral nerve sheath tumor
Meningiomas	Tumors arising from meningothelial (arachnoid) cells covering the CNS	Meninges of the brain and spinal cord	Generally favorable; some atypical or anaplastic subtypes	- Meningothelial meningioma - Fibrous meningioma - Transitional meningioma - Atypical meningioma - Anaplastic meningioma
Mesenchymal, non-meningothelial tumors involving the CNS	Diverse soft tissue tumors affecting the CNS, not originating from the meninges	Various CNS locations	Variable, depending on histology	- Hemangiopericytoma (solitary fibrous tumor) - Other sarcomas
Melanocytic tumors	Tumors derived from melanocytes in the leptomeninges	Leptomeninges and CNS surfaces	Variable, some aggressive	- Primary CNS melanoma - Melanocytoma
Haematolymphoid tumors involving the CNS	Tumors of lymphoid or hematopoietic origin involving the CNS	CNS parenchyma and meninges	Generally poor prognosis	- Primary CNS lymphoma - Leukemia involvement
Germ cell tumors	Tumors derived from germ cells, mostly in midline CNS structures	Pineal and suprasellar regions	Variable, some are highly responsive to therapy	- Germinoma - Teratoma - Yolk sac tumor - Embryonal carcinoma
Tumors of the sellar region	Tumors arising in or near the pituitary gland and sellar region	Sellar and parasellar regions	Variable, often favorable with treatment	- Pituitary adenoma - Craniopharyngioma - Pituicytoma
Metastases to the CNS	Secondary tumors from systemic cancers metastasizing to the CNS	Various CNS sites	Generally poor prognosis	- Metastases from lung cancer - Breast cancer - Melanoma - Renal cell carcinoma, etc.

Gliomas are the most common primary brain tumors and are believed to arise from neuroglial stem or progenitor cells. Histologically, they are traditionally classified into astrocytic, oligodendroglial, or ependymal subtypes and graded by the WHO from I to IV based on their degree of malignancy (Ostrom et al., 2022). Grade I gliomas, such as pilocytic astrocytomas, are typically well-circumscribed, slow-growing, and associated with a favorable prognosis. Grade II gliomas are infiltrative and exhibit low proliferative activity but tend to recur and may progress to higher grades. Grade III gliomas demonstrate increased mitotic activity and greater aggressiveness. Grade IV gliomas, exemplified by glioblastoma (GBM), are highly malignant, characterized by microvascular proliferation and necrosis, and are associated with poor clinical outcomes (WHO Classification of Tumours Editorial Board, 2021).

Gliomas are the most common type of brain tumor and cause the majority of brain tumor-related deaths. They make up nearly 30% of all primary brain tumors and account for approximately 80% of malignant cases (Weller et al., 2015). Adult-type diffuse gliomas, particularly GBM (IDH-wildtype), formerly known as glioblastoma multiforme, are the most prevalent malignant CNS tumors in adults. Despite aggressive multimodal treatment, GBM remains associated with a dismal prognosis, with a median survival of less than 15 months. In contrast, many pediatric-type low-grade gliomas exhibit favorable outcomes. Molecular alterations, including IDH mutations, 1p/19q co-deletion, H3 K27M and G34 mutations, and MAPK pathway activation, play a central role in the pathogenesis and classification of these tumors (WHO Classification of Tumours Editorial Board, 2021). Current treatment options remain largely ineffective, underscoring the urgent need for innovative and more effective therapeutic strategies (Angom et al., 2023).

Other CNS tumor types, such as ependymal tumors, are now classified not only by histological features but also by anatomical location and specific molecular profiles, reflecting a deeper understanding of their biology (WHO Classification of Tumours Editorial Board, 2021). These aggressive brain tumors typically develop in early childhood, with the highest incidence occurring in children under 4 years old (Stiller et al., 2019). Approximately one-third of cases occur in children and teenagers, with a higher prevalence in the intracranial region in this group. In contrast, spinal tumors are more common in adults, highlighting differences in tumor location and clinical presentation across age groups (Jünger et al., 2024). The 5-year survival rate varies, and many patients experience disease progression within this period. Complete surgical resection and radiation therapy are more commonly administered in pediatric patients (Elsamadicy et al., 2020).

Meningiomas (MNs) are among the most common primary intracranial tumors in adults (WHO Classification of Tumours Editorial Board, 2021). Approximately 50% of sporadic cases show biallelic somatic inactivation of the NF2 gene (Blakeley et al., 2012). MN lesions are categorized into 15 histopathological subtypes, graded from 1 to 3 based on morphological features and biological behavior. They are graded as benign (grade I; most types), potentially causing severe neurological deficits due to brain or spinal cord compression, or as atypical (grade II; e.g. chordoid and clear cell) and anaplastic (grade III) when more aggressive (e.g. papillary and rhabdoid; WHO Classification of Tumours Editorial Board, 2021). These higher-grade variants exhibit rapid growth and increased recurrence rates (Claus et al., 2005; Kleihues et al., 2002).

The standard treatment is maximal surgical resection, with radiotherapy reserved for recurrent or aggressive cases. Tumors that progress despite surgery and radiation often carry a poor prognosis (Apra et al., 2018). MNs are a hallmark feature of Neurofibromatosis type 2 (NF2), a genetic disorder characterized by mutations in the NF2 gene and the presence of bilateral vestibular schwannomas. In this context, meningiomas significantly contribute to morbidity and mortality (Angus et al., 2018), reinforcing the need for noninvasive therapies for both NF2-associated and sporadic forms.

Medulloblastomas are the most frequent malignant brain tumors in children. These embryonal tumors grow into the fourth ventricle or are located in the cerebellar parenchyma (Blaser and Harwood-Nash, 1996). Some cerebellar tumors are located laterally in a hemisphere, and almost all of these belong to the Sonic Hedgehog (SHH)-activated molecular group (Teo et al., 2013). Wingless/Int-1 (WNT)-activated medulloblastomas are thought to arise from cells in the dorsal brainstem. However, not all brainstem embryonal tumors are WNT-activated medulloblastomas (Jessa et al., 2019; Gibson et al., 2010).

They are classified in subgroups: WNT-activated, SHH-activated TP53-wildtype, SHH-activated TP53-mutant, and non-WNT/non-SHH tumors, further divided into Group 3 and Group 4 (Northcott et al., 2012). WNT-activated tumors, often occurring in older children and teenagers, typically have an excellent prognosis. In contrast, SHH-activated tumors exhibit variable outcomes depending on *TP53* status, *TP53*-wildtype cases tend to have an intermediate prognosis, whereas *TP53*-mutant tumors are associated with poor outcomes and increased treatment resistance. Group 3 tumors, often presenting with *MYC* amplification and metastatic disease, generally have the worst prognosis. Group 4, the most common subgroup, shows intermediate outcomes and is frequently associated with chromosomal alterations such as isochromosome 17q. This classification has significantly improved risk stratification and guides treatment decisions, highlighting the need for subgroup-specific therapeutic approaches (WHO Classification of Tumours Editorial Board, 2021). Advances in molecular subclassification have refined risk stratification and treatment planning, significantly improving the clinical management of these patients.

### 3.2 The role of Eph receptors and ephrin in cancer

The Ephs are classified into two main subclasses: the EphA group, comprising nine members (EphA1–A8 and EphA10), and the EphB group, which includes five members (EphB1–B4 and EphB6; Kullander and Klein, 2002). Structurally, Eph receptors consist of three distinct regions: an extracellular domain, a transmembrane segment, and an intracellular domain. The extracellular portion contains a ligand-binding domain, a cysteine-rich region, and two fibronectin type III repeats. The transmembrane domain is a short helical structure that connects the extracellular and intracellular regions across the plasma membrane. The intracellular domain encompasses a tyrosine kinase domain, a sterile alpha motif (SAM), and a PDZ-binding motif, all essential for propagating downstream signaling cascades (Himanen et al., 2001; Labrador et al., 1997).

Receptor classification is primarily based on ligand-binding preferences. Eph receptors interact with ephrins, which are also divided into two classes: ephrin-A ligands, anchored to the cell membrane through a glycosylphosphatidylinositol (GPI) linkage. Ephrin-B ligands are characterized by a transmembrane domain and a short cytoplasmic tail (Tang et al., 2020).

Typically, A-type Eph receptors bind most or all ephrin-A ligands, whereas B-type receptors preferentially engage ephrin-B ligands (Papadakos et al., 2022). Nevertheless, exceptions exist: EphA4 can bind both A-type ligands and the majority of B-type ligands. Similarly, ephrinA5 interacts not only with A-type receptors but also selectively with EphB2, showing no affinity for other EphB receptors (Papadakos et al., 2022; Surawska et al., 2004).

The Eph/ephrin interaction establishes a vital cell communication system characterized by bidirectional signaling, encompassing both forward and reverse pathways (Surawska et al., 2004). Notably, ephrin ligands can function as receptors, while Eph receptors may act as ligands. Forward signaling relies mainly on phosphoserine-dependent mechanisms, initiating multiple molecular cascades that convey signals into the cytoplasm (Surawska et al., 2004). These cascades involve key effectors such as Janus kinase (JAK)-signal transducer and activator of transcription (STAT), small GTPases from the Rho and Ras families, focal adhesion kinase (FAK), and phosphoinositide 3-kinase (PI3K; Kullander and Klein, 2002). Conversely, reverse signaling occurs within ephrin-expressing cells, where phosphorylation of tyrosine residues in the cytoplasmic tail of ephrin-B ligands triggers signal transduction and activates downstream effectors (Surawska et al., 2004).

Eph/ephrin signaling is fundamental for various developmental processes, including growth cone retraction during axon guidance, synapse formation between neurons, cell sorting in embryonic patterning, cell migration, platelet aggregation, and vascular remodeling. These pathways critically regulate cellular adhesion, motility, and proliferation (Lisabeth et al., 2013; Arvanitis and Davy, 2008).

While essential in normal physiology, the Eph family exhibits a dual role in cancer. Some studies report tumor-suppressive effects by inhibiting proliferation, invasion, and metastasis. However, accumulating evidence associates Eph receptor activity with poor prognosis, facilitating tumor progression and dissemination (Anderton et al., 2021; Surawska et al., 2004).

## 4 Eph receptors and ephrin in central nervous system tumors

### 4.1 Gliomas, glioneuronal tumors, and neuronal tumors

#### 4.1.1 EphA1 and ephrinA1

**Figure 1:**
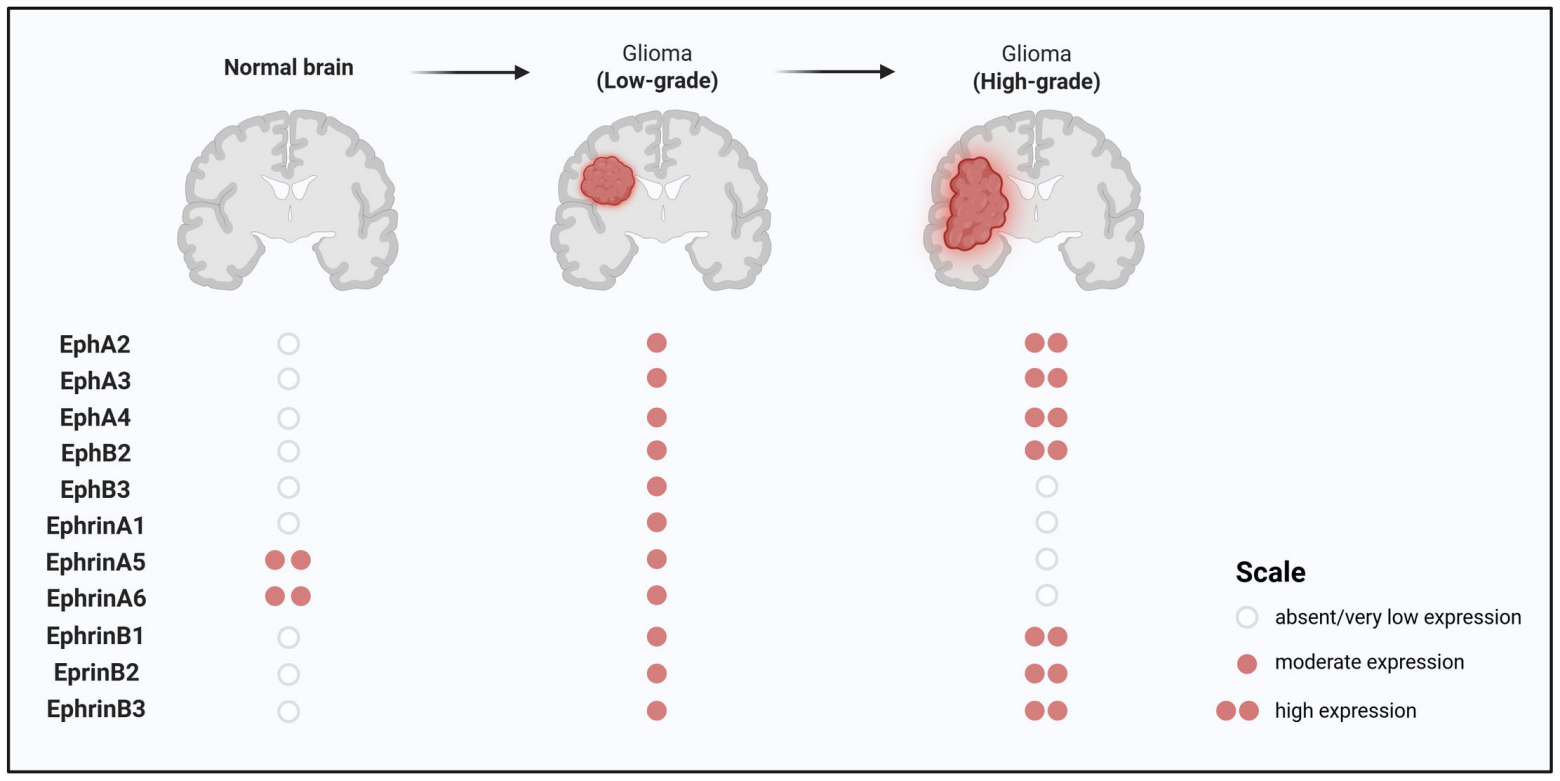
Illustration showing a brain with the medulloblastoma region highlighted. Two activation pathways are described: Sonic Hedgehog (SHH) and Non-Sonic Hedgehog (non-SHH). SHH involves EphB1, EphB4, EphrinA4, EphrinB3. Non-SHH involves EphA2, EphA8, EphB1, EphrinA1, EphrinA3, EphrinB3. It notes that the expression patterns of Eph and ephrin receptors in SHH and non-SHH medulloblastomas are linked to tumor-promoting functions and enhanced dissemination (° absent/very low expression, • moderate expression, •• high expression).

Ephrin-A1 expression is significantly downregulated in glioma cell lines and primary gliomas compared to normal brain tissue (Hao et al., 2021; Liu et al., 2007). In low-grade gliomas, higher ephrin-A1 levels are associated with increased infiltration of CD4^+^ T cells, myeloid dendritic cells, and neutrophils. Additionally, tumors with high ephrin-A1 expression are enriched for genes involved in the intrinsic apoptotic pathway, suggesting a tumor-suppressive and immunomodulatory role (Hao et al., 2021; Table 2; Figure 1).

**Table 2:** Eph/ephrin signaling in CNS tumors.

**Eph/ephrin**	**Tumor type(s)**	**Prognostic value**	**Main effects**	**Therapeutic strategies**
**Gliomas, glioneuronal tumors, and neuronal tumors**				
*EphA1/ephrinA*	Gliomas (low- and high-grade)	Positive	Suppresses tumor growth and migration via EphA2-FAK inhibition	Targeted cytotoxins, adenoviral vectors, nanoparticles, hMSC-based gene therapy
*EphA2/ephrinA2*	Gliomas and GBM,	Negative	Promotes invasion, stemness, angiogenesis; inhibited by ephrin-A1	RNA aptamers, small molecules (GLPG1790), CAR T cells, bispecific antibodies, EphA2 blockers
*EphA3/ephrinA3*	GBM	Negative	Sustains stemness via MAPK; supports immune evasion and invasion	CAR T cells, cytotoxic fusion proteins, multivalent drug conjugates, nanoparticles
*EphA4/ephrinA4*	Gliomas, GBM	Negative	Enhances MAPK/Akt signaling; pro-apoptotic action suppressed through interaction with ephrin-B3	Disruption of EphA4/ephrin-B3 axis (e.g., ephrin-B3 silencing)
*EphA5/ephrinA5*	Gliomas	Downregulated: Negative Upregulated: Positive	Ephrin-A5 suppresses tumorigenicity by promoting c-Cbl-mediated EGFR degradation; Epigenetic repression by Bmi1 enhances proliferation and invasion via H3K27me3 at the ephrin-A5 promoter	Epigenetic modulation (e.g., Bmi1 inhibition), ephrin-A5 mimetics
*EphA6/ephrinA6*	GBM	Positive	Enhances BMP-2-induced apoptosis in glioma-initiating cells	BMP-sensitizing therapies via ephrin-A6
*EphA7/ephrinA7*	GBM	Negative	Associated with tumor aggressiveness and angiogenesis	Prognostic marker to select patients for tyrosine kinase inhibitor therapy
*EphB1/ephrinB1*	Glioma; GBM	EphrinB1: Negative EphB1: Positive	Enhances proliferation and migration; associated with immune modulators and oncogenic signaling; Acts as a tumor suppressor; inhibits ephrin-B2-induced invasion and migration, lacks detectable phosphorylation in tumor cells	Silencing ephrin-B1 reduces tumor aggressiveness, a potential target for immunomodulatory or invasion-inhibiting therapies; Overexpression or activation of EphB1 could be explored to suppress tumor invasion; a potential therapeutic sensitizer
*EphB2/ephrinB2*	Gliomas, GBM; ependymomaa	Negative	Promotes invasion (via R-Ras/paxillin), EMT, and endothelial interaction; Promotes stemness and blocks differentiation	Inhibit ephrin-B2, EphB2-R-Ras targeting, activate EphB4 axis; Potential target for Ras/MAPK pathway modulation
*EphB3/ephrinB3*	Gliomas, GBM	Negative	EphB3 suppresses EGFR/PI3K/AKT, ephrinB3 promotes invasion via Rac1	Boost EphB3 or block ephrin-B3-Rac1 interaction
*EphB4/ephrinB4*	Gliomas	Positive: Neoplastic cells; Negative: microenvironment (blood vessels)	Inhibits tumor cell migration and invasion, and blocks the Akt signaling pathway; Remodeling of blood vessels due to altered interactions between pericytes and endothelial cells, resulting in wide vasculature and resistance to treatment, highly expressed in poorly differentiated tumors	Use of ephrin-B2 agonists to activate EphB4, dual targeting of tumor cells and vasculature to reduce resistance and aggressiveness
*EphB6/ephrinB6*	Gliomas, GBM	Negative	Induces tumor-specific CD8^+^ CTLs; immunogenic secretory variant	Peptide vaccines, UniPR1331 (pan-Eph inhibitor), combination with Bevacizumab
**Embryonal tumors**				
*EphA1/ephrinA1*	Medulloblastoma (non-SHH)	Unclear	Minimal basal activation; limited responsiveness to ephrin-A1 stimulation; slight activation by ephrin-B1, indicating receptor promiscuity; May contribute to subtype-specific signaling phenotypes	No specific therapies reported
*EphA2/ephrinA2*	Medulloblastoma (Groups 3 and 4)	Negative	Enhances the angiogenic potential of EphA2; supports vascularization in aggressive MB subtypes	Potential for anti-EphA2 or anti-angiogenic therapy
*EphA3/ephrinA3*	Medulloblastoma (non-SHH)	Unclear	High expression; possible role in development	No specific therapies reported
*EphA4/ephrinA4*	Medulloblastoma (SHH)	Unclear	Low EphA4 levels; no significant effect on tumor growth in knockout mouse models; potential functional role in SHH-driven MB	No specific therapies reported
*EphA5/ephrinA5*	Medulloblastoma	Negative	Promotes tumor growth via Akt; high in fetal brain/tumor	Genetic or pharmacologic inhibition of ephrin-A5 shows therapeutic promise
*EphA6/ephrinA6*	Medulloblastoma (Subtype D)	Potentially positive	Correlates with genes linked to axonal guidance and neuronal differentiation; potentially influencing migration and tumor progression	Potential target for modulating differentiation/migration
*EphA7/ephrinA7*	Medulloblastoma	Unclear	Low EphA7 level; no significant effect on tumor growth in knockout mouse models; Potential functional role in SHH-driven MB	No specific therapies reported
*EphA8/ephrinA8*	Medulloblastoma (C, D, E subtypes)	Negative	Implicated in neuronal migration pathways; overexpressed in tumor tissue	No specific therapies reported
*EphB1/ephrinB1*	Medulloblastoma (SHH and non-SHH)	Negative	High EphB1 expression is associated with increased aggressiveness, therapy resistance; Promotes migration, invasion, cell cycle progression, and therapy resistance	targeted inhibition of EphB1 to reduce migration and improve radiosensitivity
*EphB2/ephrinB2*	Medulloblastoma	Negative	Promotes tumor invasion and migration; Activates PI3K-Akt-mTOR and Ras-Raf-MEK-Erk; Phosphorylation is stimulated by ephrin-B1 and ephrin-B2; Regulates cell motility via multiple signaling nodes	Targeting receptors and ligands may reduce invasion
*EphB3/ephrinB3*	Medulloblastoma (SHH and non-SHH)	Potentially positive	Lower EphB3 levels may correlate with more aggressive or invasive tumor phenotypes, Expression decreases in migratory and less stem-like tumor cell populations; EprhinB3 likely supports normal Eph receptor signaling dynamics without actively driving tumor progression	Enhancing EphB3 function could suppress tumor invasiveness
*EphB4/ephrinB4*	Medulloblastoma (SHH)	Potentially positive	Lower EphB4 expression is associated with increased invasion and possibly with more aggressive or stem-like tumor behavior; EphB4 activation appears to be tightly regulated and possibly linked to SHH signaling pathways	Enhancing EphB4 function could suppress tumor invasiveness
**Meningiomas**				
*EphA2, A4*, and *B1* and *ephrin-A2, A4*, and *B1*	NF2-deficient meningiomas	Negative	Activation of EphA2 and EphB1, along with Src/SFK signaling; crosstalk with mTORC1/2 and ERBB3; promotes growth and survival	Dasatinib (inhibits EphA2/B1 and Src); Combined inhibition of mTORC1/2 and Src/SFK pathways; Dual blockade of mTORC1/2 and IGF1R/insulin signaling for enhanced growth suppression

Functional studies support this interpretation. In U251 glioma cells, forced expression of ephrin-A1 significantly inhibited migration, proliferation, and anchorage-independent growth. These effects agreed with decreased levels of EphA2 and its downstream effector FAK, indicating that ephrin-A1 suppresses glioma progression via inhibition of the EphA2-FAK signaling axis (Liu et al., 2007).

Therapeutic strategies have been developed to harness ephrin-A1's antitumor properties. One study used bacterial cytotoxins fused to ephrin-A1 and IL-13 mutants, targeting EphA2 and IL13RA2 via convection-enhanced delivery in dogs with intracranial gliomas. The approach achieved broad intratumoral distribution (median coverage: 70%; range: 40–94%), was well tolerated, and produced objective tumor responses in 50% (8/16) of cases, with tumor volume reductions of up to 94% (Rossmeisl et al., 2021).

Another study used an adenovirus vector delivering an ephrinA1-PE38 fusion protein with GM-CSF elicited strong antitumor activity in glioma-bearing rats. Treatment reduced tumor volume, extended survival, and activated dendritic cells, suggesting the creation of *in situ* dendritic cell vaccines. This strategy offers localized and systemic immune responses while avoiding the limitations of *ex vivo* dendritic cell manipulation (Li et al., 2015b).

Building on this, chitosan-coated nanoparticles delivering the same ephrinA1-PE38/GM-CSF construct showed enhanced dendritic cell activation, tumor eradication, and prolonged survival in glioma models, highlighting their promise for personalized immunotherapy (Li et al., 2015a).

Finally, human mesenchymal stem cells (hMSCs) engineered to secrete ephrinA1-PE38 selectively targeted EphA2-overexpressing glioma cells. Intratumoral injection of these hMSCs suppressed tumor growth *in vivo*, supporting stem cell-based gene delivery as a viable and targeted treatment strategy for malignant gliomas (Sun et al., 2011).

#### 4.1.2 EphA2 and ephrinA2

EphA2 is overexpressed in approximately 67% of glioma patients and is inversely correlated with overall survival, making it a key prognostic marker alongside VEGF and vWF (Shen et al., 2021). Its expression increases with tumor grade and predicts poor prognosis, while higher levels of ephrin-A1 are associated with lower-grade tumors. Patients with EphA2-positive/ephrin-A1-negative tumors have significantly worse overall and progression-free survival (Li et al., 2010).

In glioma stem cells (GSCs), EphA2 promotes invasion through Akt-mediated phosphorylation at serine 897. This signaling remains active without ligand binding but is suppressed by ephrin-A ligands. In EphrnA1/3/4 triple-knockout mice, GSC invasion increased, while EphA2 knockdown reduced self-renewal, stemness, and tumorigenicity. Notably, EphA2 enhances stem-like traits independent of its kinase activity, while disruption of the Akt-EphA2 axis impairs neurosphere formation (Miao et al., 2015).

Immunohistochemical studies in glioma patients showed significantly higher expression of EphA2 (90.91%) and MMP-2 (86.36%) in high-grade tumors compared to low-grade cases. MRI features such as peritumoral edema, contrast enhancement, and tumor size also correlated with EphA2 and MMP-2 expression, reinforcing their role in glioma invasiveness (Suo et al., 2019).

**Figure 2:**
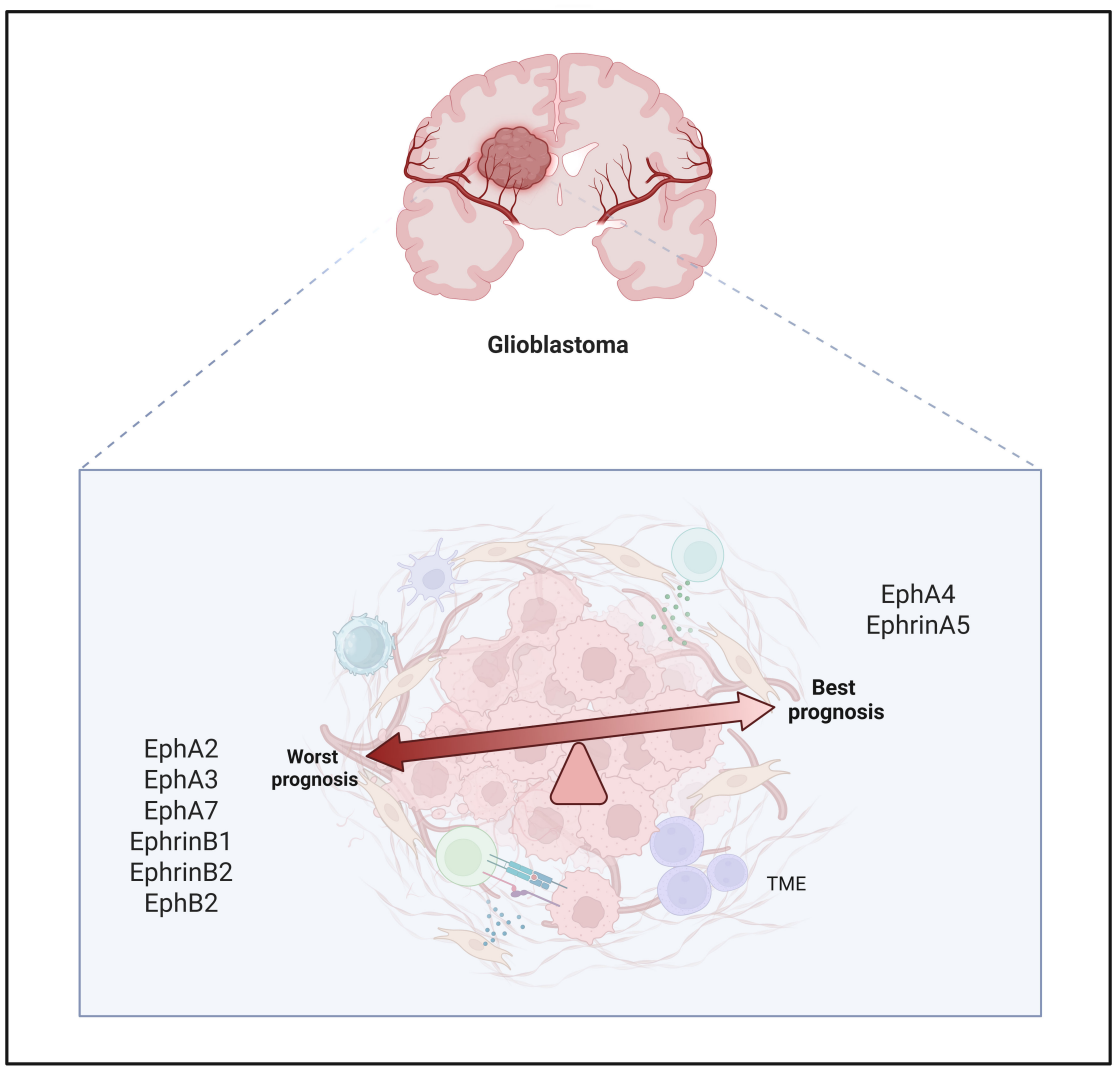
Association between Eph/ephrin expression and prognosis in glioblastoma. The heterogeneous expression of Eph receptors and ephrin ligands within the glioblastoma tumor microenvironment (TME) shows that the high levels of EphA2, EphA3, EphA7, EphrinB1, EphrinB2 and EphB2 are associated with poor prognosis. Conversely, the elevated expression of EphA4 and EphrinA5 correlates with better prognosis, suggesting potential opposing roles of different Eph/ephrin family members in glioblastoma biology.

EphA2 is overexpressed in roughly 90% of GBM cases and is significantly elevated compared to normal brain tissue and lower-grade gliomas. In contrast, ephrin-A1 levels are often low. Activation of EphA2 by ephrin-A1 suppresses anchorage-independent growth and invasion in a dose-dependent manner, confirming its oncogenic role and therapeutic potential (Wykosky et al., 2005; Figure 2).

Within GBM tumor-propagating cells (TPCs), EphA2 expression correlates with higher tumorigenicity. Knockdown of EphA2 or treatment with ephrinA1-Fc impairs self-renewal and tumor growth (Binda et al., 2012). In recurrent GBM, coexpression of EphA2 and EphA3 marks a highly tumorigenic GSC subpopulation. Dual knockdown promotes differentiation and prevents tumor formation, while a bispecific antibody targeting both receptors significantly reduces tumor burden (Qazi et al., 2018).

EphA2 also interacts with integrin α3 at the cell periphery in U251MG cells, contributing to migration and adhesion via focal adhesion complexes (Makarov et al., 2013). Functionally, EphA2 acts downstream of MEK/ERK/RSK signaling, where EGF induces its S897 phosphorylation. This is blocked by MEK or RSK inhibitors and reduced by ephrin-A1 or EphA2 knockdown (Hamaoka et al., 2016). Interestingly, a Wnt5a^∧^High/EphA2^∧^Low GSC subpopulation was more tumorigenic than Wnt5a^∧^Low/EphA2^∧^High cells, and combined inhibition of Wnt5a and EphA2 drastically reduced invasion and tumor growth (Trivieri et al., 2022).

Several therapeutic approaches have been developed to target EphA2. Filamin A forms a complex with EphA2, promoting its phosphorylation and GBM cell proliferation. Silencing filamin A blocks EGF-induced activation and tumor growth (Tamura et al., 2022). RNA aptamers such as 40L and A40s selectively bind EphA2, inhibiting GSC proliferation and migration. A40s are particularly promising due to their serum stability and ability to cross the blood-brain barrier (Affinito et al., 2020).

The small-molecule inhibitor GLPG1790 blocks EphA2 phosphorylation at Tyr588 and Ser897, modulates differentiation markers, and reduces tumor growth, showing synergy with standard therapies (Gravina et al., 2019).

EphA2-directed CAR T cells enhance anti-tumor responses by increasing IFN-γ and OX40 expression, and their efficacy improves further when combined with PD-1 blockade (An et al., 2021). Finally, UniPR1454, a novel l-β-homotryptophan derivative, inhibits EphA2/ephrin-A1 interaction and significantly reduces GBM cell proliferation (Guidetti et al., 2024).

#### 4.1.3 EphA3 and ephrinA3

EphA3, a receptor tyrosine kinase, is frequently overexpressed in gliomas, especially in GBM and its mesenchymal subtype. It is enriched in tumor-initiating cells, where it helps maintain an undifferentiated, stem-like state by modulating MAPK signaling. Suppressing EphA3, via gene silencing or radiolabeled monoclonal antibodies, significantly reduces tumorigenicity (Day et al., 2013).

Targeting EphA3 with CAR T cells has shown potent and specific cytotoxicity against GBM and diffuse midline glioma (DMG) cells *in vitro*. In orthotopic xenograft models, these cells eradicate tumors and induce durable immune memory. Notably, mice rechallenged with tumors on the contralateral side remained tumor-free for over 6 months, underscoring the therapeutic potential of EphA3-directed immunotherapy (Lertsumitkul et al., 2024).

EphA3 is detected in up to 60% of GBM samples and is particularly enriched in invasive fronts, perivascular niches, and tumor-infiltrating immune cells. A cytotoxic fusion protein based on ephrin-A5, which targets EphA3, EphA2, and EphB2, selectively kills GBM cells with remarkable potency (IC_50_ ≈ 10^−11^ M; Ferluga et al., 2016).

Further validating its therapeutic relevance, EphA3-directed CAR T cells generated from healthy donors effectively eliminate EphA3-positive GBM cells, patient-derived neurospheres, and organoids. These CAR T cells produce curative responses in orthotopic GBM models. Complementing this approach, a multivalent conjugate (QUAD 3.0) targeting EphA3, EphA2, EphB2, and IL13RA2 was developed. Linked to doxorubicin derivatives, this construct binds all four receptors with nanomolar affinity and delivers cytotoxic agents to tumor cells with minimal toxicity *in vivo* (Sharma et al., 2020).

To bypass the blood-brain barrier, temozolomide-conjugated gold nanoparticles functionalized with anti-EphA3 antibodies have been engineered for intranasal delivery. This formulation improves drug uptake, enhances apoptosis in glioma cells, and lowers IC50 by 18.5-fold in TMZ-resistant GBM models, significantly extending survival in treated animals (Wang et al., 2021).

#### 4.1.4 EphA4 and ephrinA4

EphA4 mRNA is expressed at levels four times higher in gliomas than in normal brain tissue. In U251 cells, EphA4 promotes proliferation and migration by enhancing FGF2-induced MAPK and Akt signaling and activating Rac1/Cdc42. Its physical interaction with FGFR1 further amplifies these oncogenic pathways (Fukai et al., 2008).

Also, EphA4 acts as a dependence receptor, inducing apoptosis in the absence of its ligand ephrin-B3. In GBM, elevated ephrin-B3 expression suppresses this pro-apoptotic signal, promoting the survival of both tumor and endothelial cells. Silencing ephrin-B3 reduces angiogenesis and tumor growth, highlighting the therapeutic potential of disrupting the EphA4/ephrin-B3 axis (Royet et al., 2017).

#### 4.1.5 EphA5 and ephrinA5

Ephrin-A5 is significantly downregulated in primary gliomas. Its forced expression in U373 cells suppresses tumorigenicity by promoting c-Cbl-mediated ubiquitination and degradation of EGFR, leading to reduced receptor levels. Similar effects are achieved using ephrinA5-Fc or EphA2-Fc, reinforcing its tumor-suppressive role (Li et al., 2009).

This suppression is epigenetically regulated. Bmi1 represses ephrin-A5 by inducing H3K27me3 enrichment at its promoter region, thereby enhancing GBM cell proliferation and invasion. This mechanism is active in glioma-initiating cells (GICs), suggesting that restoring ephrin-A5 signaling or targeting the Bmi1-ephrinA5 axis may represent a promising therapeutic strategy for Bmi1-overexpressing GBMs (Ricci et al., 2020).

#### 4.1.6 EphA6 and ephrinA6

Ephrin-A6 is downregulated in GBM but correlates with improved patient prognosis. Although bone morphogenetic protein (BMP) signaling promotes differentiation and apoptosis in glioma-initiating cells (GICs), some subpopulations are resistant. Ephrin-A6 enhances BMP-2-induced apoptosis in both BMP-sensitive and -resistant GICs, highlighting its therapeutic potential in overcoming BMP resistance (Raja et al., 2019).

#### 4.1.7 EphA7 and ephrinA7

Elevated EphA7 protein expression in GBM is associated with poor clinical outcomes, especially when accompanied by high microvascular density. Immunohistochemical evaluation of EphA7 offers valuable prognostic insight and may serve as a surrogate marker to identify patients who could benefit from tyrosine kinase inhibitor therapy (Wang et al., 2008).

#### 4.1.8 EphB1 and ephrinB1

Ephrin-B1 is consistently upregulated in gliomas, where it promotes tumor progression by enhancing proliferation, migration, and invasion. Its expression correlates with poor prognosis and advanced disease stages. Silencing ephrin-B1 suppresses these malignant traits, underscoring its functional relevance. Beyond its role in tumor cell behavior, ephrin-B1 is associated with immunological changes in the tumor microenvironment, including increased infiltration of Th2 cells, macrophages, and plasmacytoid dendritic cells (pDCs). It is also linked to oncogenic pathways such as cell cycle regulation, protein processing, and viral infection responses (Zheng and Shi, 2025).

In GBM, high ephrin-B1 expression independently predicts poor overall survival. A prognostic model incorporating ephrin-B1-associated immune modulators effectively stratifies patient outcomes. Functionally, ephrin-B1 silencing reduces GBM cell proliferation and migration, further supporting its oncogenic and immunomodulatory roles in high-grade gliomas (Shi et al., 2021).

In contrast, EphB1 appears to play a tumor-suppressive role. Although EphB1 expression levels are similar across glioma grades, only EphB1 is associated with improved survival in malignant gliomas. Mechanistically, EphB1 lacks detectable tyrosine phosphorylation in glioma cells and, when overexpressed, inhibits ephrin-B2-induced migration and invasion both *in vitro* and *in vivo* (Teng et al., 2013).

#### 4.1.9 EphB2 and ephrinB2

EphB2 is overexpressed in glioma cells and plays a central role in tumor invasion and migration. In U87 cells, it localizes to lamellipodia during migration and promotes motility when activated. Its overexpression in U251 cells enhances invasion and reduces cell adhesion, confirming its pro-invasive function. These effects are mediated through R-Ras signaling, which regulates adhesion, proliferation, and invasion. Silencing R-Ras blocks EphB2 activity, and its phosphorylation correlates with tumor grade and EphB2 expression, reinforcing this invasive pathway (Nakada et al., 2004).

EphB2 is also stabilized under hypoxia by HIF-2α and promotes GBM cell invasion via paxillin phosphorylation, suggesting a HIF-2α-EphB2-paxillin axis that supports epithelial-mesenchymal transition (EMT) and tumor aggressiveness (Qiu et al., 2019). The ephrin-B2 ligand is equally critical, particularly in glioma stem-like cells (GSCs), where it drives perivascular invasion and homotypic migration via RhoA activation. Upregulation of ephrin-B2 enhances tumor initiation, while its inhibition, genetic or antibody-mediated, significantly impairs tumor progression in preclinical models (Krusche et al., 2016).

Clinically, ephrin-B2 expression correlates with high tumor grade and lower Karnofsky performance scores, serving as an independent prognostic marker for shorter progression-free survival (Tu et al., 2012). Similarly, ephrin-B2 mRNA levels are elevated in GBM compared to normal brain, and its expression is higher in invasive cell lines like U87. Functional studies show that ephrin-B2 promotes migration and invasion, and its blockade significantly suppresses these malignant behaviors (Nakada et al., 2004).

In GBM, ephrin-B2 is not only upregulated but also highly phosphorylated, indicating active signaling. It localizes to both tumor cells and vasculature, and endothelial-specific deletion reduces tumor growth and perfusion, mimicking antiangiogenic therapy. However, no additive benefit is seen when combined with VEGFR2 inhibition, suggesting shared angiogenic pathways (Broggini et al., 2022).

Interestingly, ephrin-B2 knockdown in GBM xenografts paradoxically increases tumor growth. This is reversed by ephrin-B2-Fc treatment, which activates EphB4 signaling. EphB4 activation inhibits proliferation and invasion, indicating a complex ephrin-B2-EphB4 regulatory axis that could be exploited therapeutically through simultaneous ephrin-B2 inhibition and EphB4 stimulation (Bhatia et al., 2020).

Moreover, in mouse models, EphB2 overexpression in Ink4a/Arf(–/–) neural stem cells induces ependymoma formation by enhancing proliferation, suppressing differentiation, and activating Ras and p38 MAPK signaling, highlighting its role in maintaining a stem-like, tumorigenic phenotype (Chen et al., 2015).

#### 4.1.10 EphB3 and ephrinB3

EphB3 expression inversely correlates with glioma aggressiveness, decreasing progressively with tumor grade. In glioma cell lines such as U87MG and U251, low EphB3 levels are associated with increased proliferation, migration, and invasion, primarily through upregulation of EGFR and activation of the PI3K/AKT signaling pathway. Conversely, EphB3 overexpression suppresses these malignant traits by downregulating this pathway, indicating its tumor-suppressive role and highlighting its potential as a diagnostic and therapeutic target (Xiao et al., 2024).

Ephrin-B3 plays a complementary pro-invasive role. It is upregulated in migrating cells from various glioma lines and invading cells from patient-derived biopsies. In functional studies, ephrin-B3 overexpression in low-expressing cell lines (U87, T98G) promotes invasion and migration, while its knockdown in high-expressing lines (U251, SNB19) reduces motility and disrupts Rac1-dependent lamellipodia formation. Notably, ephrin-B3 is essential for EphB2/Fc-induced invasion, reinforcing its role in glioma cell motility. In patient tumors, ephrin-B3 expression and phosphorylation levels correlate with histological grade and are enriched in invasive glioblastoma cells (Nakada et al., 2006).

#### 4.1.11 EphB4 and ephrin-B4

EphB4 acts as a negative regulator of glioma progression. Stimulation of glioma cells with their specific ligand, ephrin-B2, induces EphB4 phosphorylation, which suppresses migration, invasion, and Akt signaling. These inhibitory effects are abolished by EphB4 silencing, underscoring its functional role in restraining malignancy. Immunostaining studies show EphB4 expression is localized to the tumor core, while ephrin-B2 is more widely distributed, suggesting that EphB4-ephrin-B2 interactions may help retain tumor cells centrally and limit peripheral invasion (Kawahara et al., 2019).

Beyond its role in cell motility, EphB4 also contributes to glioma vascular remodeling. Its overexpression leads to the development of large, treatment-resistant blood vessels by altering pericyte-endothelial interactions and vascular architecture. Co-localization of EphB4/ephrin-B2 with CD34 in stromal microvessels and GFAP in tumor cells has been observed, with higher expression levels in poorly differentiated tumors, reinforcing its relevance to both tumor aggressiveness and vascular pathology (Uhl et al., 2018; Xiao et al., 2004).

#### 4.1.12 EphB6 and ephrin-B6

EphB6 is preferentially expressed in malignant gliomas, and a unique secretory variant, undetectable in normal tissue, has been identified by RT-PCR in most glioma cell lines. This isoform includes a distinct 54-amino acid sequence, from which peptides were shown to bind HLA-A2402 and elicit peptide-specific CD8^+^ cytotoxic T lymphocytes (CTLs) in HLA-A24^+^ glioma patients. These CTLs exert HLA-A24-restricted cytotoxicity, positioning EphB6 variant peptides as promising candidates for peptide-based immunotherapy in this subgroup of patients (Jin et al., 2008).

Complementing this immunotherapeutic potential, UniPR1331, a pan-Eph receptor antagonist, demonstrates strong anti-angiogenic and anti-tumor activity in GBM xenograft and orthotopic models. Treatment significantly reduces tumor volume and extends disease-free survival. When combined with Bevacizumab, UniPR1331 shows enhanced therapeutic efficacy, highlighting the value of Eph-targeted strategies in glioblastoma treatment (Festuccia et al., 2018).

### 4.2 Medulloblastoma

#### 4.2.1 EphA1 and ephrin-A1

EphA1 expression is reduced in medulloblastoma cell lines (DAOY, Res-220, Res-256, Uw-426, Uw-473, Uw-402) compared to normal fetal brain and adult cerebellum. Phosphorylation analysis revealed minimal EphA1 activation under basal conditions, with only a slight increase observed in Uw-402 cells. Stimulation with ephrin-A1 failed to elicit notable phenotypic effects, whereas ephrin-B1 induced modest activation of type A Eph receptors in Uw-402, suggesting cross-reactivity and ligand promiscuity within the Eph/ephrin system in medulloblastoma (Sikkema et al., 2012).

**Figure 3:**
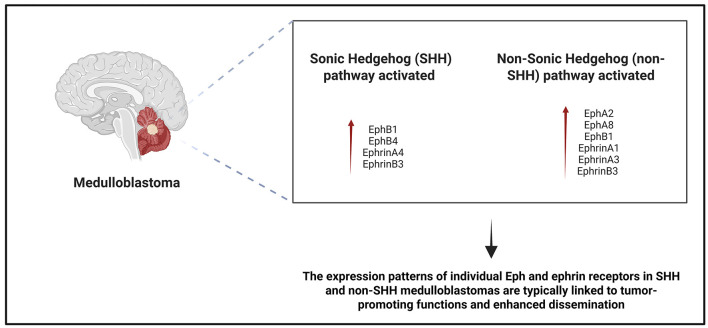
Differential expression profiles of Eph receptors and ephrin ligands in medulloblastomas with activation of the sonic hedgehog (SHH) or non-SHH signaling pathways. Increased expression of EphB1, EphB4, ephrinA4 and ephrinB3 was observed in SHH-driven tumors. Non-SHH medulloblastomas show increased expression of EphA2, EphA8, EphB1, ephrinA1, ephrinA3, and ephrinB3. These expression patterns are generally associated with tumor-promoting functions, including increased dissemination and aggressiveness.

Furthermore, ephrin-A1 expression appears largely confined to non-SHH medulloblastoma subtypes, with minimal or absent expression in SHH-driven tumors. This subtype-specific expression pattern suggests a potential role for ephrin-A1 in non-SHH medulloblastoma biology, although its functional significance remains unclear (McKinney et al., 2015; Figure 3).

#### 4.2.2 EphA2 and ephrin-A2

EphA2 is highly expressed in medulloblastoma cell lines Res-300 and Uw-426, with moderate phosphorylation detected in Uw-402 following stimulation (Sikkema et al., 2012). Although EphA2 signaling is generally associated with tumor-suppressive effects, its activation in this context may paradoxically promote angiogenesis via VEGF-A and TNF, likely influenced by high ephrin-A1 expression in endothelial cells. Supporting this, microarray data from 29 medulloblastoma samples revealed subgroup-specific expression patterns: EphA2 is enriched in non-SHH tumors (WHO groups 3 and 4) but absent in SHH subtypes, suggesting a potential role in the pathobiology of more aggressive medulloblastoma variants (Morrison et al., 2013).

#### 4.2.3 EphA3 and ephrin-A3

EphA3 expression is elevated in primary medulloblastomas compared to normal cerebellum, with the highest levels observed in the Uw-402 cell line and normal fetal brain, suggesting its involvement in both neural development and tumor biology (Sikkema et al., 2012). Similarly, ephrin-A3 is predominantly expressed in non-SHH medulloblastomas, showing diffuse immunopositivity in over 95% of confirmed cases. Although ephrin-A3 may serve as a secondary ligand for receptors like EphB1, there is currently no direct evidence supporting its oncogenic activity (McKinney et al., 2015).

#### 4.2.4 EphA4 and ephrin-A4

EphA4 expression is consistently reduced in medulloblastoma cell lines compared to normal brain tissue (Sikkema et al., 2012). However, knockout studies in mouse models lacking EphA4 and EphA7 revealed no significant impact on tumor size, suggesting that these receptors may not play a central role in tumor growth (Bhatia et al., 2015). In contrast, ephrin-A4 is predominantly expressed in SHH-subtype medulloblastomas, indicating a possible subtype-specific function that remains to be clarified (Bhatia et al., 2015).

#### 4.2.5 EphA5 and ephrin-A5

EphA5 is highly expressed in the Uw-402 medulloblastoma cell line and normal fetal brain, suggesting a role in both cerebellar development and tumorigenesis (Sikkema et al., 2012). In ND2-SmoA1 transgenic mice, loss of ephrin-A5—along with EphA4 and EphA7—significantly reduced tumor size and p-Akt levels. Larger tumors in wild-type mice showed increased p-Akt and PCNA, implicating ephrin-A5 as a promoter of medulloblastoma growth via Akt pathway activation. Notably, only complete ephrin-A5 deletion suppressed tumor progression (Bhatia et al., 2015).

#### 4.2.6 EphA6 and ephrin-A6

EphA6 exhibits subtype-specific expression in medulloblastoma, particularly enriched in subtype D, where it correlates with genes linked to axonal guidance and neuronal differentiation, such as Ephrin-B1, RND1, RND2, and SEMA3A. These associations suggest a role for EphA6 in regulating neuronal architecture and cellular organization within the tumor microenvironment, potentially influencing migration and tumor progression (Gokhale et al., 2010). However, expression analyses in medulloblastoma cell lines revealed reduced ephrin-A6 levels compared to normal tissue, implying suppression of its homeostatic neuronal functions in the malignant setting (Sikkema et al., 2012).

#### 4.2.7 EphA7 and ephrin-A7

EphA7 expression is markedly reduced in medulloblastoma cell lines relative to normal tissue, indicating a distinct role in tumor biology (Sikkema et al., 2012). In double knockout mice lacking EphA4 and EphA7 (EphA4^−^/^−^ EphA7^−^/^−^ Smo), MRI and Western blot analyses showed no consistent changes in tumor size, despite correlations between tumor volume, p-Akt, and PCNA levels. The inability to generate EphA7 single knockout mice due to severe phenotypes limits further functional studies. These findings suggest that EphA7, a high-affinity receptor for ephrin-A5, may have redundant roles with other Eph receptors in medulloblastoma progression, though its exact function remains to be clarified (Bhatia et al., 2015).

#### 4.2.8 EphA8 and ephrin-A8

EphA8 is significantly overexpressed in primary medulloblastoma tissues compared to normal cerebellum, suggesting a role in tumor-specific biology (Sikkema et al., 2012). Multiple studies have linked EphA8 expression to medulloblastoma subtypes associated with neuronal migration pathways and poor prognosis. Gokhale et al. (2010) and Kool et al. (2008) identified its expression mainly in subtypes C and D, while Kool also reported involvement in subtype E—subgroups typically classified as non-WNT/non-SHH tumors. Supporting this, McKinney et al. (2015) observed EphA8 expression to be largely restricted to non-SHH subtypes, reinforcing its relevance to their molecular landscape, although its precise functional contribution remains to be elucidated.

#### 4.2.9 EphB1 and ephrin-B1

EphB1 is frequently expressed in medulloblastoma, particularly in SHH and non-SHH subtypes, with over 90% of tumors showing diffuse immunopositivity. Its expression is prominent in DAOY and D556 cell lines and absent in fetal cerebellum, indicating a tumor-specific profile (McKinney et al., 2015).

Functionally, EphB1 promotes tumor aggressiveness. In DAOY and UW228 cells, siRNA-mediated knockdown reduced EphB1 expression, leading to impaired migration, downregulation of β1-integrin and p-Src, increased G1 arrest, and enhanced radiosensitivity. Silencing EphB1 also delayed tumor recurrence after irradiation, indicating its role in cell cycle progression and resistance to therapy (Bhatia et al., 2015).

Transcriptomic analyses revealed EphB1 enrichment in tumor spheres with high self-renewal and in migrating cells, supporting its role in motility and stemness (Morrison et al., 2013). EphB1 is also upregulated in subtype D medulloblastomas, which are characterized by axon guidance and neuronal differentiation signatures, suggesting its involvement in tumor plasticity (Gokhale et al., 2010).

EphB1 is co-expressed with genes such as ephrin-B1, RND1/2, and SEMA3A, key regulators of neuronal migration, synaptogenesis, and axonal development, indicating that neurodevelopmental pathways may be hijacked to support tumor growth (Gokhale et al., 2010; Kool et al., 2008).

Among these genes, ephrin-B1 plays a key role by activating EphB1 independently of Src. Its overexpression increases EphB1/B2 phosphorylation, remodels the cytoskeleton, reduces adhesion, enhances proliferation, and alters migratory behavior. Ephrin-B1 knockdown impairs migration, potentially via STAT3 or Par-6 signaling, pointing to non-canonical pathways underlying its oncogenic effects (McKinney et al., 2015).

Ephrin-B1 is enriched in highly proliferative, dense tumor regions but absent in the fetal cerebellum, reinforcing its tumor-specific function. Functionally, ephrin-B1 modulates multiple pathways, including p38, MSK1/2, Stat1/2/4/5a/6, and paxillin, following ligand stimulation or EphB2 inhibition, suggesting broad influence over tumor behavior (Sikkema et al., 2012).

Despite its generally high levels of expression, some studies report reduced EphB1 levels in specific medulloblastoma lines (DAOY, Res-220, UW-426) compared to normal cerebellum, suggesting context-dependent regulation or inhibitory mechanisms within certain tumor environments (Sikkema et al., 2012).

#### 4.2.10 EphB2 and ephrinB2

EphB2 is upregulated in primary medulloblastomas compared to normal cerebellum, with high expression observed in DAOY and Res-300 cell lines. Functionally, ephrin-B1, but not ephrin-A1, enhanced invasion in DAOY and, to a lesser extent, in UW-402 cells, which express intermediate levels of EphB2. No effect was seen in Res-256 cells with low EphB2 expression. Ephrin-B1 also reduced adhesion to collagen (DAOY and UW-402) and laminin (DAOY only). These effects were reversed by EphB2 knockdown, confirming its role in promoting invasiveness and reducing adhesion (Sikkema et al., 2012).

Mechanistically, ephrin-B1 stimulation increased EphB2 phosphorylation and activated PI3K-Akt-mTOR and Ras-Raf-MEK-Erk pathways. In contrast, EphB2 silencing elevated phosphorylation of Erk1/2, mTOR, p27, and paxillin, indicating that EphB2 tightly regulates cell motility through multiple signaling nodes. These findings position EphB2 as a promising therapeutic target to limit medulloblastoma invasion. Additional studies support EphB2's relevance. Kool et al. (2008) confirmed its overexpression in tumors relative to normal cerebellum, and Morrison et al. (2013) showed EphB2 transcript enrichment in migrating DAOY cells but downregulation in self-renewing tumor spheres, highlighting its specific role in motility over stemness.

In a larger cohort, EphB2 was expressed in over 95% of tumors, with its phosphorylation dependent on ephrin-B1 and ephrin-B2 co-activation (McKinney et al., 2015). While tumor-specific and active in forward signaling, EphB2 does not appear to function as a primary oncogenic driver, suggesting that targeting both receptor and ligand may be necessary to disrupt its pro-invasive effects.

Ephrin-B2 contributes to this axis, with elevated expression in DAOY cells and the fetal brain compared to adult cerebellum (Sikkema et al., 2012). Detected in over 95% of medulloblastomas and fetal Purkinje cells, ephrin-B2 shows diffuse immunopositivity. Although its knockdown significantly reduced EphB1/B2 and Src phosphorylation, it did not affect proliferation, suggesting that ephrin-B2 supports classical Eph signaling without directly driving oncogenesis (McKinney et al., 2015).

#### 4.2.11 EphB3 and ephrinB3

EphB3 expression is consistently downregulated in medulloblastoma. qPCR analysis revealed reduced EphB3 levels in migratory cells compared to central tumor regions, and in low vs. high self-renewing tumor spheres, suggesting a context-dependent, potentially suppressive role within the Eph-ephrin axis (Morrison et al., 2013). This downregulation is further supported by decreased EphB3 mRNA levels across multiple medulloblastoma cell lines (DAOY, Res-220, Res-256, UW-426, UW-473, and UW-402) relative to fetal brain and adult cerebellum. Ephrin-B3, its putative ligand, shows higher expression in DAOY cells and fetal brain, and is present in 70-90% of SHH and non-SHH medulloblastomas (Sikkema et al., 2012). While its expression is relatively widespread, it has not been directly linked to oncogenic activity. This suggests ephrin-B3 may contribute to basal signaling dynamics rather than actively driving tumor progression (McKinney et al., 2015).

#### 4.2.12 EphB4 and ephrinB4

Similar to EphB3, EphB4 expression is downregulated in migratory medulloblastoma cells, suggesting an inverse relationship with invasion and a possible association with stem-like tumor phenotypes (Morrison et al., 2013). Among medulloblastoma cell lines, EphB4 expression was higher in DAOY and UW-426, whereas Res-256 exhibited minimal basal phosphorylation. Notably, ephrin-B1 stimulation significantly increased EphB4 phosphorylation in DAOY cells, indicating that its activation is ligand-dependent (Sikkema et al., 2012).

Microarray profiling of 29 medulloblastomas, validated in an additional cohort of 60 tumors, revealed that EphB4 is predominantly expressed in SHH-subtype tumors, with minimal expression in non-SHH variants. This pattern suggests a potential link between EphB4 and Sonic Hedgehog signaling, although its precise role in medulloblastoma biology remains to be defined (McKinney et al., 2015).

### 4.3 Meningiomas

#### 4.3.1 EphA2, A4, and B1 and ephrin-A2, A4, and B1 signaling

In NF2-null meningioma cell lines, kinome profiling revealed activation of Eph receptor tyrosine kinases (EphA2, EphB1), c-KIT, and the Src/SFK pathway, targets of the FDA-approved kinase inhibitor Dasatinib. These cells showed increased expression and phosphorylation of EphA2 and EphB1, along with elevated Src/SFK (Y416) phosphorylation. Dasatinib treatment (2h) significantly reduced phosphorylation of EphA2 (S897), EphB1 (Y594), and Src/SFK (Y416), without affecting mTORC1/2 activity. Transcriptomic analysis also showed elevated EphB1 and EphA4 expression. Variability in pEphA2, pEphB1, and pSrc/SFKs among tumors indicated intertumoral heterogeneity. Combined Dasatinib and dual mTORC1/2 inhibition led to stronger growth suppression, suggesting co-targeting Eph RTK/SFK and mTORC1/2 pathways may benefit NF2-deficient meningiomas (Angus et al., 2018).

Another study showed that NF2 loss activates autocrine NRG1-ERBB3 signaling, which interacts with EphA2 and mTORC1/2. In NF2-null human arachnoidal and Ben-Men-1 cells, NF2 deficiency increased NRG1 secretion and ERBB3 activation, promoting crosstalk with EphA2 and mTORC1/2. mTORC1/2 inhibition suppressed NRG1/ERBB3 signaling but triggered compensatory pAkt T308 activation via the IGF1R/insulin receptor. Dual blockade of mTORC1/2 and IGF1R/insulin signaling effectively reduced pAkt T308 and cell viability, supporting co-inhibition as a potential therapeutic strategy in NF2-deficient meningiomas (Beauchamp et al., 2021).

## 5 Adverse effects and toxicity associated with targeting Eph/ephrin signaling

The current evidence base provides only limited insights into safety of Eph/ephrin inhibitors. All reports are preclinical and focus predominantly on antitumor efficacy, with systematic toxicology rarely assessed or reported.

Across murine glioblastoma models, administration of the pan-Eph/ephrin inhibitor UniPR1331 produced modest thrombocytopenia alongside minor adaptive changes in hepatic and renal parameters. Although not clearly severe in animals, such findings underscore the importance of incorporating routine hematologic and biochemical monitoring into GLP toxicology assessments, particularly given the role of Eph/ephrin signaling in vascular and hematopoietic homeostasis (Festuccia et al., 2018).

Similarly, studies of the GLPG1790, a selective inhibitor of EPHA2, reported minimal observable toxic effects, although no specific toxicology experiments were undertaken. In comparative models, conventional therapies such as radiotherapy and temozolomide demonstrated substantially greater cytotoxicity, reflected in higher tissue necrosis (Gravina et al., 2019).

In addition, intracranial administration of the QUAD 3.0-WP936, a multivalent vector protein that can target four receptors: EphA3, EphA2, EphB2, and also IL-13RA2, at escalating doses (167 nM−1.7 μM) in C57BL/6 mice did not impair motor activity, grooming behavior, or body weight, suggesting absence of overt neurological toxicity at tested concentrations (Sharma et al., 2020).

In summary, the safety data remain limited to a small number of preclinical reports that, while describing modest hematologic, renal and hepatic changes, lack systematic evaluation. Consequently, there is a need for well-designed, rigorous preclinical studies to define safety margins, followed by clinical trials.

## 6 Future research and clinical translation of Eph-signaling in CNS tumors

Eph/ephrin signaling is broadly dysregulated across primary central nervous system tumors, but its functional consequences are highly context- and lineage-dependent, varying with tumor type, molecular subgroup, cellular state and the microenvironment (Liu et al., 2007; Hao et al., 2021; Sikkema et al., 2012; McKinney et al., 2015; Morrison et al., 2013; Bhatia et al., 2015; Gokhale et al., 2010; Kool et al., 2008). In diffuse gliomas, loss or reduction of putative suppressive ligands such as ephrin-A1 and ephrin-A5 is common and, when retained, these ligands associate with immune infiltration and a pro-apoptotic regulatory network, which suppresses malignant behaviors by inhibiting the EphA2-FAK axis and similar signaling pathways (Liu et al., 2007; Hao et al., 2021; Rossmeisl et al., 2021; Li et al., 2015b,a; Sun et al., 2011; Li et al., 2009).

Conversely, high-grade gliomas, most strikingly GBM, frequently co-opt Eph receptors and ephrin-B ligands to sustain invasion, stemness and aberrant vascular phenotypes. EphA2 is upregulated with grade, promotes ligand-independent, Akt-mediated invasive signaling and supports glioma stem cell self-renewal and tumorigenicity, while EphA3 is enriched in tumor-initiating and mesenchymal compartments and is targetable with CAR T cells and ligand-toxin constructs in preclinical models (Shen et al., 2021; Li et al., 2010; Miao et al., 2015; Suo et al., 2019; Wykosky et al., 2005; Binda et al., 2012; Qazi et al., 2018; Day et al., 2013; Lertsumitkul et al., 2024; Ferluga et al., 2016; Sharma et al., 2020; Wang et al., 2021).

The ephrin-B2-EphB2 axis is a recurrent driver of perivascular invasion, EMT-like programmers and angiogenesis in GBM, and perturbation of this axis produces complex, sometimes paradoxical, outcomes that reflect bidirectional and context-specific signaling (Nakada et al., 2004; Qiu et al., 2019; Krusche et al., 2016; Tu et al., 2012; Broggini et al., 2022; Bhatia et al., 2020; Chen et al., 2015). Other Eph family members display mixed roles: EphA4 can both potentiate oncogenic FGF-Rac1 signaling and function as a dependence receptor neutralized by ephrin-B3; ephrin-A5 and ephrin-A6 exhibit tumor-suppressive associations through EGFR degradation and sensitization to BMP-induced apoptosis; and receptors such as EphB3 and EphB4 appear reduced in migratory or stem-like compartments, consistent with context-dependent suppressive roles (Fukai et al., 2008; Royet et al., 2017; Li et al., 2009; Ricci et al., 2020; Raja et al., 2019; Xiao et al., 2024; Nakada et al., 2006; Kawahara et al., 2019; Uhl et al., 2018; Xiao et al., 2004). Taken together, these findings emphasize intratumorally heterogeneity in gliomas and the need to interpret Eph/ephrin biology in the light of subgroup, ligand availability, phosphorylation state, and co-activated pathways (Liu et al., 2007; Hao et al., 2021; Shen et al., 2021; Li et al., 2010; Miao et al., 2015; Suo et al., 2019; Wykosky et al., 2005; Binda et al., 2012; Qazi et al., 2018; Li et al., 2009; Ricci et al., 2020; Raja et al., 2019; Nakada et al., 2004; Qiu et al., 2019; Krusche et al., 2016; Tu et al., 2012; Broggini et al., 2022).

Medulloblastoma exhibits a similarly complex, subgroup-specific Eph/ephrin landscape that nevertheless differs in important ways from diffuse gliomas. Several A-class receptors (EphA2, EphA3, EphA5, EphA8) and B-class receptors (EphB1, EphB2) are upregulated in subsets of tumors, particularly within non-SHH subgroups, while others (EphA1, EphA4, EphA7, EphB3, EphB4) are often reduced or display context-dependent downregulation (Sikkema et al., 2012; McKinney et al., 2015; Morrison et al., 2013; Bhatia et al., 2015; Gokhale et al., 2010; Kool et al., 2008). Functionally, EphB1 and EphB2 emerge as mediators of motility, invasion and therapeutic resistance: EphB1 knockdown impairs migration, reduces β1-integrin and p-Src, induces G1 arrest and increases radio sensitivity; EphB2 activation promotes invasion through PI3K-Akt-mTOR and Ras-Raf-MEK-ERK signaling and reduces cell-matrix adhesion (Sikkema et al., 2012; McKinney et al., 2015; Morrison et al., 2013; Bhatia et al., 2015).

Ligand distribution and receptor promiscuity further shape outcomes, ephrin-A and B ligands show subgroup-restricted patterns (for example ephrin-A4 in SHH tumors and ephrin-A3/A1 enrichment in non-SHH tumors), and ephrin-B1 co-activation of EphB receptors is particularly important for cytoskeletal remodeling and invasion (Sikkema et al., 2012; McKinney et al., 2015; Morrison et al., 2013; Bhatia et al., 2015). Genetic models corroborate ligand dependence: complete loss of ephrin-A5 reduces tumor size and downstream Akt signaling in mice, yet redundancy among family members and severe phenotypes in certain knockouts (e.g., difficulty isolating EphA7 single knockouts) caution that simple loss-of-function interpretations may be misleading and demand careful *in vivo* study design (Bhatia et al., 2015). Because medulloblastoma subgroups differ in development and microenvironmental wiring, the Eph/ephrin system there argues strongly for subgroup-stratified target selection and for prioritizing receptor-ligand pairs with validated functional impact (Sikkema et al., 2012; McKinney et al., 2015; Morrison et al., 2013; Bhatia et al., 2015; Gokhale et al., 2010; Kool et al., 2008).

Also, NF2-deficient meningiomas provide a complementary example of how tumor genetics rewires Eph-linked networks and mandates combination approaches. Kinome profiling of NF2-null meningioma models revealed activation of Eph receptors (EPHA2, EPHB1), c-KIT and Src family kinases that are inhibited by dasatinib, but mTORC1/2 signaling remained active and limited monotherapy efficacy; parallel studies showed NF2 loss drives autocrine NRG1-ERBB3 signaling that crosstalk with EphA2 and mTORC1/2, and that mTORC1/2 inhibition triggers compensatory pAkt T308 activation via IGF1R/insulin receptor which is only abrogated by combined mTORC1/2 and IGF1R/insulin blockade (Angus et al., 2018; Beauchamp et al., 2021). These observations exemplify a recurrent translational principle: single-node inhibition frequently unmasks adaptive, compensatory circuits that blunt efficacy, arguing for rational co-targeting strategies guided by molecular profiling.

Therapeutic implications across these tumor types are convergent. First, mono-target approaches risk failure because of ligand redundancy, receptor promiscuity and adaptive signaling; second, GBM in particular requires strategies that simultaneously address invasion, stemness and vascular support while overcoming central nervous system delivery barriers. Rational modalities include receptor-directed biologics (bispecific antibodies, CAR T cells), high-affinity ligand-toxin conjugates and selective small molecules, deployed with advanced delivery platforms (nanoparticles, intranasal routes, cell-based vectors) and combined with inhibitors of co-activated downstream networks where evidence supports such pairing (Tamura et al., 2022; Affinito et al., 2020; Gravina et al., 2019; An et al., 2021; Guidetti et al., 2024; Day et al., 2013; Lertsumitkul et al., 2024; Ferluga et al., 2016; Sharma et al., 2020; Wang et al., 2021; Nakada et al., 2004; Qiu et al., 2019; Krusche et al., 2016; Tu et al., 2012; Broggini et al., 2022; Bhatia et al., 2020; Chen et al., 2015; Xiao et al., 2024). For medulloblastoma, subgroup stratification is essential: interventions should prioritize receptor-ligand pairs and pathways validated within the specific molecular subgroup (Sikkema et al., 2012; McKinney et al., 2015; Morrison et al., 2013; Bhatia et al., 2015; Gokhale et al., 2010; Kool et al., 2008).

Safety and translational readiness must be foregrounded. Many preclinical studies lack systematic GLP toxicology and report safety heterogeneously, which impairs risk assessment. Because Eph receptors and ephrins are expressed in normal neural, endothelial and perivascular compartments and contribute to angiogenesis, blood-brain-barrier integrity and neural development, modulation of these pathways, especially with pan-Eph inhibitors, potent cytotoxins or systemic biologics, carries biologically plausible on-target risks that require careful toxicological characterization and early clinical monitoring (Kullander and Klein, 2002; Surawska et al., 2004; Papadakos et al., 2022; Lisabeth et al., 2013).

## 7 Conclusion

The Eph/ephrin signaling axis plays a multifaceted and context-dependent role in CNS tumors. Overall, the Eph/ephrin system emerges as a key regulator of tumor behavior across CNS malignancies. Its diverse roles, ranging from developmental mimicry to promotion of invasion and therapeutic resistance, highlight its potential as a biomarker and therapeutic target. Future studies should focus on elucidating context-specific signaling mechanisms and on developing dual-targeting strategies that address both Eph receptors and their ephrin ligands to enhance therapeutic precision and efficacy.
